# London’s liaison psychiatry services: survey of service provision

**DOI:** 10.1192/pb.bp.114.046862

**Published:** 2015-04

**Authors:** Smitha Naidu, Jim Bolton, Jared Smith

**Affiliations:** 1 South West London & St George’s Mental Health NHS Trust; 2 St Helier Hospital, Surrey; 3 St George’s, University of London

## Abstract

**Aims and method** To describe the liaison psychiatry services of all 30 general hospitals in Greater London and to determine whether services met national recommendations. The results were compared with a similar survey conducted 8 years previously to determine whether there had been significant service development.

**Results** We identified wide variations in service provision across London. Fifteen hospitals (50%) had 24-hour services and one had no service. There had been a significant increase in services that assessed older adults. Increases in the size of teams and consultant psychiatry staff were not significant.

**Clinical implications** Despite an increasing emphasis on the effectiveness of liaison psychiatry services, no London hospital had staffing levels consistent with national recommendations. Recent evidence for the cost-effectiveness of liaison psychiatry and an emphasis on parity between physical and mental health in National Health Service policy may provide further impetus for growth.

Liaison psychiatry is concerned with the management of mental disorder in general medical settings where there are high rates of mental health problems. Mental disorder accounts for 5% of emergency department attendances; 30% of hospital in-patients have comorbid mental illness.^1^

A comprehensive liaison psychiatry service will address the following clinical needs:^1^

- patients presenting at the emergency department with mental health needs;
- comorbid mental and physical disorders;
- patients being treated for the physical complications of alcohol and substance misuse;
- where physical illness and its treatment is causing mental health problems;
- medically unexplained physical symptoms.

In addition, liaison psychiatry services have a role in the training of general medical staff in the recognition and basic management of common mental health problems.^2^

The benefits of a comprehensive liaison psychiatry service for a general hospital fall into four key domains:^3^

1. improved psychiatric and medical outcomes of patients
2. enhanced patient experience of medical care
3. increased patient safety
4. greater cost-effectiveness of medical services.

The National Health Service (NHS) Confederation highlighted the economic benefits of liaison psychiatry services, which are primarily achieved by decreasing the length of hospital stays and reducing the frequencies of reattendance and readmission.^4^ A subsequent economic analysis of a 24-hour liaison psychiatry service found that it generated considerable cost savings for the health economy, with a cost–benefit ratio of 4:1.^5^ The greatest cost benefit was found in service provision for older adults.

Following increased recognition of the clinical and economic benefits of liaison psychiatry services, the Academy of Medical Royal Colleges recommended the provision of 24-hour multidisciplinary liaison psychiatry services for emergency departments and in-patient wards.^6^ In addition, the need for specific liaison psychiatry service provision for older adults has been emphasised.^7,8^

To meet a need for more explicit guidance on the provision of services for patients with mental health problems in general hospital settings, the Royal College of Psychiatrists^9^ updated its recommendations for the staffing of liaison psychiatry services (Table 1). These were reiterated in national commissioning guidelines.^1^ The need for this guidance arose, in part, from the recognition of the wide variability in service provision.

In 2004, a survey of liaison psychiatry services in 29 general hospitals across Greater London identified wide variations in staffing, working hours and patient groups seen.^10^ Although half of services worked over 24 hours, all except one service fell short of national recommendations for service provision. Similar deficits have been identified in other areas of the UK.^11–13^

Following a national focus on emergency care, there had been an expansion in liaison psychiatry services serving emergency departments. However, there was concern that

**Table 1:** Summary of liaison psychiatry staffing recommendations^9^^,^^a^

Role	Whole time equivalents
Consultant psychiatrist	1.0

Trust grade doctor	1.0

Nurses	5.0

Clinical health psychologist	1.0

Administrator	1.5

a. These recommendations are for a service operating from Monday to Friday, 09.00 h to 17.00 h, assessing and managing adults of all ages in a 650-bed general hospital. Psychiatric training posts are not included and are in addition to the staff above.

services for other general hospital patients might be neglected as a result.

In light of recent recommendations for the establishment of robust liaison psychiatry services, this survey aimed to identify changes in service provision across London over the 8 years up to mid-2012, and to audit the staffing of these services against national standards.

## Method

Greater London comprises 32 London boroughs and the City of London, and has 30 general hospitals with emergency departments. Information on bed numbers was obtained from hospital websites.

An email and telephone survey of liaison psychiatry services was carried out over the first 6 months of 2012. A senior clinician from each of the services was asked a list of predetermined questions. We enquired about the number and professions of clinical team members. Higher specialist trainees in psychiatry were not included in these figures as such posts are often supernumerary and may not continue beyond the current post-holder’s attachment.

We established details of service delivery. Hours of work were categorised into services operating within core ‘working hours’ (09.00 h to 17.00 h, Monday to Friday), those delivering an extended-hours service and those operating 24 hours per day.

**Table 2:** Comparison of the 2004 and 2012 profiles of the directly comparable liaison psychiatry services (*n* = 27)

Service variable	2004	2012	2004 *v.* 2012 *P*
Number of in-patient beds, mean (s.d.)	638 (232)	530 (242)	0.001

Number of whole time equivalent staff, mean (s.d.)	8.4 (6.0)	9.0 (5.7)	0.63

Hours of service, *n* (%)			
No service	0 (0.0)	1 (3.7)	
Working hours (09.00 h to 17.00 h)	5 (18.5)	6 (22.2)	
Extended hours	9 (33.3)	6 (22.2)	
24 hours	13 (48.1)	14 (51.9)	0.80

Staffing, *n* (%)			
Dedicated medical psychiatry staff	19 (70.4)	23 (85.2)	0.06
Dedicated consultant psychiatry staff	19 (70.4)	23 (85.2)	0.06

Patient groups seen, *n* (%)			
Older adults	17 (63.0)	26 (96.3)	0.01
Alcohol and substance misuse	21 (77.8)	23 (85.2)	0.55

The survey enquired about service provision for the following specific patient groups:

- those presenting to the emergency department
- in-patients
- out-patients
- older adults
- those with alcohol and/or substance misuse
- those with perinatal mental health problems.

These groups were selected as being those most commonly served by a comprehensive liaison psychiatry service. Where specialist liaison teams existed to manage specific patient groups (e.g. older adults), these were included within the data collected for the overall liaison psychiatry service. Information was also collected on the organisations responsible for the funding and management of services.

The results of the survey were analysed using the Statistical Package for the Social Sciences, Release 19.0 (on IBM). Staffing levels were compared with the Royal College of Psychiatrists’ recommendations (Table 1). Following reconfiguration of acute hospital services between 2004 and 2012, we judged that differences between liaison psychiatry staffing and service provision in these 2 years could be compared at 27 sites.^10^ Data from the two surveys were compared using the Wilcoxon Signed-Rank Tests for continuous variables and the McNemar (mid-*p*) test for categorical variables, which is appropriate for binary matched pairs data with small and moderate sample sizes.^14^ The criterion for statistical significance was set at *P*<0.05.

## Results

### Hospitals

Information was collected from all 30 hospitals, of which 29 had a liaison psychiatry service. Between 2004 and 2012, 2 hospitals had closed and 3 new sites had opened; 27 hospitals were common to both surveys.

A comparison of the profiles of the 27 directly comparable services is given in Table 2.

### Bed numbers

The mean number of in-patient beds for the 30 hospitals was 535 (range 200–1200, s.d. = 235). For the 27 comparable sites there was a significant decrease in bed numbers of 17% over the previous 8 years (*P* = 0.001).

### Working hours

Six (20%) hospitals had services operating in core working hours (09.00 h to 17.00 h, Monday to Friday). Eight (27%) hospitals had extended-hours services and 15 (50%) had 24-hour services. At the 27 comparable sites, there was no significant change in the hours of work between 2004 and 2012 (*P* = 0.80).

In the 15 hospitals with either no liaison psychiatry service or where the service operated for less than 24 hours, out-of-hours cover by community mental health services was available at 13 sites (87%).

### Patient groups

Table 3 describes the patient groups assessed by services and indicates where a particular group was managed by a specific specialist team within the overall liaison psychiatry service.

All of the 29 services assessed patients in the hospital’s emergency department. One service only assessed patients of 65 years of age or over; younger adults were referred to community mental health services.

All of the liaison teams accepted referrals for older adults and 14 (48%) had a specific specialist older adults service. For the 27 comparable sites there was a significant increase in liaison psychiatry service provision for older adults between 2004 and 2012 (*P* = 0.006), but not for patients with alcohol and substance misuse (*P* = 0.55).

### Staffing

The mean number of whole time equivalent clinical staff for all 29 teams was 8.7 (range 1–22, s.d. = 5.5). The mean numbers of staff for the various hours of service are given in Table 4.

With respect to the 27 directly comparable sites, there had not been a statistically significant increase in the mean size of teams (*P* = 0.63).

Three teams (10%) consisted solely of nursing staff, but had access to senior medical staff if required. Fourteen teams (48%) had at least one whole time equivalent consultant psychiatrist. Two teams (7%) had a whole time

**Table 3:** Patient groups managed by liaison psychiatry services in London’s general hospitals (*n* = 30)

Patient groups	Liaison psychiatry service *n* (%)	Specialist service provision within the liaison service *n* (%)
Emergency department	29 (97)	0 (0)

In-patients	28 (93)	2 (7)

Out-patients	16 (53)	1 (3)

Older adults	29 (97)	14 (48)

Alcohol and substance misuse	26 (87)	10 (33)

Perinatal	26 (87)	9 (30)

**Table 4:** Staffing of London’s liaison psychiatry services (*n* = 29)

	Whole time equivalent number of staff, mean (s.d.)				
Hours of service	Consultant psychiatrist	Other medical	Nursing	Psychology	Other
Working hours (*n* = 6)	0.8 (0.5)	0.8 (0.7)	1.8 (1.0)	0.0 (0.0)	0.0 (0.0)

Extended hours (*n* = 8)	0.5 (0.4)	1.3 (1.0)	5.1 (4.7)	0.3 (0.4)	0.2 (0.5)

24 hours (*n* = 15)	0.9 (0.7)	1.5 (1.4)	8.4 (2.4)	0.1 (0.2)	0.4 (1.1)

equivalent psychologist and five more (17%) had regular psychology sessions.

At the directly comparable sites there had been an increase in the number of teams with dedicated medical psychiatry staff and specifically consultant psychiatry staff, but the differences were not statistically significant (both *P* = 0.06).

National staffing recommendations for liaison psychiatry services (Table 1) are for a working-hours service, although it is noted that an extended-hours service with additional staffing should be provided where there is local need. It is difficult to compare the services surveyed against these recommendations, because of the range of different hours of work. However, none of the services employed all of the recommended staff.

### Funding and management

In total, 16 liaison psychiatry services (55%) were funded via a mental health trust, 6 (21%) via an acute trust and 7 (24%) were jointly funded. All services were managed by mental health trusts.

## Discussion

This survey of London’s general hospitals describes the level of liaison psychiatry service provision in 2012 and compares this with 8 years previously. As in 2004, the survey found a wide variation in staffing and hours of work. No hospitals had staffing levels consistent with national recommendations. Between 2004 and 2012 there was a significant increase in service provision for older adults. There was a non-significant increase in the number of liaison psychiatry teams with dedicated medical staff and consultant psychiatrists.

There continued to be considerable gaps in service provision, with one hospital having no liaison psychiatry service. Although community mental health services often provide psychiatric input where no liaison psychiatry service exists, this is likely to be a less clinically and cost-effective model of care.

The variation in service provision between hospitals has been found in surveys of other areas of the UK.^11–13^ As service provision in London has previously been found to be more extensive than elsewhere, this survey indicates that considerable development is required across the UK to fulfil national recommendations and achieve potential cost savings for the wider health economy.^5^

The increase in specific service provision for older adults might reflect the emphasis on providing such services following the previous survey.^7^ Subsequent evidence of their cost-effectiveness may provide further impetus for the growth of such services.^5^

There was an indication that psychiatric expertise within liaison psychiatry services may be increasing, including a growth in consultant numbers, although these findings did not reach statistical significance. This potential increase may reflect recognition of the need for robust clinical leadership and management, and of the specific expertise that psychiatry can bring to the management of complex cases.^15^

The decrease in mean bed numbers for London’s hospitals may reflect the emphasis in health service policy for England and Wales on providing more services in the community. If this trend continues, it could have a significant impact on how liaison psychiatry services are delivered. One potential area of service development is the extension of liaison psychiatry expertise into primary care to support the management of patients with comorbid physical and mental illness and those with medically unexplained symptoms.^15,16^

At the time of this survey, the principle of ‘parity of esteem’ between mental and physical health services was stated in England’s NHS Mandate.^17^ NHS England’s objective is to close the health gap between people with mental health problems and the population as a whole. The potential impact of this on liaison psychiatry has been articulated in a subsequent report, which recommends that commissioners need to regard liaison services as a necessity rather than an optional luxury, in order to provide an integrated approach to healthcare in acute settings.^18^

Potential changes in the funding and commissioning of liaison psychiatry services may also provide an impetus for service development. As indicated by this survey, most services in England and Wales are currently paid for from a mental health block contract.^19^ Separate funding of physical and mental health services is inappropriate for liaison psychiatry, which bridges the two areas.^3^ Work is underway to devise a sustainable model of funding that will provide more incentive for commissioners and providers of healthcare to establish comprehensive liaison psychiatry services.

### Limitations

The survey was conducted in 2012, and several respondents indicated that local commissioners were considering an increase in liaison psychiatry service provision, often on a trial basis. Hence, although at the time of publication there may have already been an increase service provision in London, it will be several years before it can be determined whether this has been sustained. We anticipate that this survey will provide a baseline for a future survey to identify the effect of an increased focus on liaison psychiatry service provision in commissioning guidance.

The survey is likely to underestimate overall mental health service provision for adults in general hospitals. We did not include stand-alone specialist services that operated separately from the main liaison psychiatry service (e.g. neuropsychiatry, psycho-oncology, clinical health psychology). Also, we did not enquire about child and adolescent liaison psychiatry services, which usually operate separately from adult services.

### Implications

The survey describes the persistent variation in liaison psychiatry service provision to London’s general hospitals, with services universally falling below recommended standards. Since the survey was undertaken, a number of national reports have highlighted the clinical and economic benefits of liaison psychiatry and emphasised the importance of parity between physical and mental health services. As well as describing recent changes in services, the survey provides a basis for future research to determine whether current recommendations are translated into the commissioning of comprehensive liaison psychiatry services for all of London’s general hospitals.
